# Evaluating Augmented Depression Therapy (ADepT): study protocol for a pilot randomised controlled trial

**DOI:** 10.1186/s40814-019-0438-1

**Published:** 2019-04-27

**Authors:** Barnaby D. Dunn, Emily Widnall, Nigel Reed, Rod Taylor, Christabel Owens, Anne Spencer, Gerda Kraag, Gerjo Kok, Nicole Geschwind, Kim Wright, Nicholas J. Moberly, Michelle L. Moulds, Andrew K. MacLeod, Rachel Handley, David Richards, John Campbell, Willem Kuyken

**Affiliations:** 1 Mood Disorders Centre, University of Exeter, Exeter, UK; 2 College of Medicine and Health, University of Exeter, Exeter, UK; 3 Faculty of Psychology and Neuroscience, Maastricht University, Maastricht, Netherlands; 4 Department of Clinical Psychological Science, Faculty of Psychology and Neuroscience, Maastricht University, Maastricht, Netherlands; 5 School of Psychology, The University of New South Wales, Sydney, Australia; 6 Department of Psychology, Royal Holloway University of London, London, UK; 7 Department of Psychiatry, University of Oxford, Oxford, UK

**Keywords:** Augmented Depression Therapy, Cognitive Behavioural therapy, Major depressive disorder, Feasibility study, Pilot study, Mixed methods

## Abstract

**Background:**

While existing psychological treatments for depression are effective for many, a significant proportion of depressed individuals do not respond to current approaches and few remain well over the long-term. Anhedonia (a loss of interest or pleasure) is a core symptom of depression which predicts a poor prognosis but has been neglected by existing treatments. Augmented Depression Therapy (ADepT) has been co-designed with service users to better target anhedonia alongside other features of depression. This mixed methods pilot trial aims to establish proof of concept for ADepT and to examine the feasibility and acceptability of a future definitive trial evaluating the clinical and cost-effectiveness of ADepT, compared to an evidence-based mainstream therapy (Cognitive Behavioural Therapy; CBT) in the acute treatment of depression, the prevention of subsequent depressive relapse, and the enhancement of wellbeing.

**Methods:**

We aim to recruit 80 depressed participants and randomise them 1:1 to receive ADepT (15 weekly acute and 5 booster sessions in following year) or CBT (20 weekly acute sessions). Clinical and health economic assessments will take place at intake and at 6-, 12-, and 18-month follow-up. Reductions in PHQ-9 depression severity and increases in WEMWBS wellbeing at 6-month assessment (when acute treatment should be completed) are the co-primary outcomes. Quantitative and qualitative process evaluation will assess mechanism of action, implementation issues, and contextual moderating factors. To evaluate proof of concept, intake-post effect sizes and the proportion of individuals showing reliable and clinically significant change on outcome measures in each arm at each follow-up will be reported. To evaluate feasibility and acceptability, we will examine recruitment, retention, treatment completion, and data completeness rates and feedback from patients and therapists about their experience of study participation and therapy. Additionally, we will establish the cost of delivery of ADepT.

**Discussion:**

We will proceed to definitive trial if any concerns about the safety, acceptability, feasibility, and proof of concept of ADepT and trial procedures can be rectified, and we recruit, retain, and collect follow-up data on at least 60% of the target sample.

**Trial registration:**

ISCRTN85278228, registered 27/03/2017

**Electronic supplementary material:**

The online version of this article (10.1186/s40814-019-0438-1) contains supplementary material, which is available to authorized users.

## Background

Depression is a debilitating, chronically recurring, and common condition (UK point prevalence 2.6%; estimated lifetime risk 35%) [1–3]. It is a significant predictor of suicide, working days lost, and poor physical health, accounting for 17% of UK disability and predicted to become the leading worldwide contributor to disability by 2020 [4–7]. Depression is estimated to cost the UK economy £12 billion annually [8] and is not satisfactorily treated by current psychosocial/pharmacological treatments (only 50% response rates; amongst responders 50% relapse within 2 years) [9, 10]. It remains unclear if existing treatments lead to markedly improved outcomes relative to placebo control conditions, except for the most severe cases [11–13]. There is a pressing need to improve treatment outcomes, both to reduce symptom severity and the disability burden associated with the disorder. One way to improve outcomes is to target features of depression that have been neglected in existing treatments but are clinically important (i.e. they predict depression prognosis and are judged by clients as key elements to repair to lead to full recovery).

One such feature of depression is anhedonia, defined as a loss of interest or pleasure in normally enjoyable activities [14]. Anhedonia is one of the two cardinal symptoms required to be diagnosed with depression. It is part of broader disturbances in depression of the positive valence system (PVS) [15] which regulates reward-seeking, consummatory behaviour and reward/habit learning in positive motivational contexts and helps shape a broader sense of wellbeing (experiencing positive mood, having meaning and purpose, and feeling socially connected) [16].

Anhedonia is a prevalent feature of depression, with approximately 95% of affected individuals reporting some loss of interest or pleasure [17] and approximately 35% showing severe anhedonic symptoms [18]. Anhedonia symptoms and broader wellbeing deficits predict depression onset, treatment response, and recurrence [19–21]. Service users report that repairing anhedonia/wellbeing is a key component of recovery, over and above reduction in other depressive symptoms [22, 23]. Despite the importance of PVS deficits, they are not explicitly targeted by mainstream treatments for depression [19–21]. For example, Cognitive Behavioural Therapy (CBT) [24] targets changing negative cognitions to reduce negative mood (i.e. the negative valence system; NVS) [15] and pays less attention to repairing anhedonia/wellbeing (i.e. the PVS). Basic science findings demonstrate that the NVS and PVS are partly dissociable [25–27]. Therefore, treatments primarily reducing one will not necessarily build the other [19].

Innovative treatments need to be developed that simultaneously target reducing negativity and building positivity [19, 20, 28]. Simultaneously targeting positive and negative valence system deficits has the potential to improve acute treatment outcomes, protect individuals from subsequent relapse, enhance recovery, and reduce the disability burden from depression.

Augmented Depression Therapy (ADepT) has therefore been developed to simultaneously target positive and negative valence system deficits, following guidance regarding the development of complex interventions [29]. ADepT design was informed by (i) translating findings from basic science research characterising PVS deficits in depression (cf., [30]) and (ii) co-designing the intervention with service users and other stakeholders to maximise subsequent uptake [31–33]. The design process followed the principles of Intervention Mapping [34]. An iterative process of stakeholder consultation, literature review, and analysis of the local context was conducted to develop the ADepT protocol, updating a logic model of the intervention as we went.

**Fig. 1:**
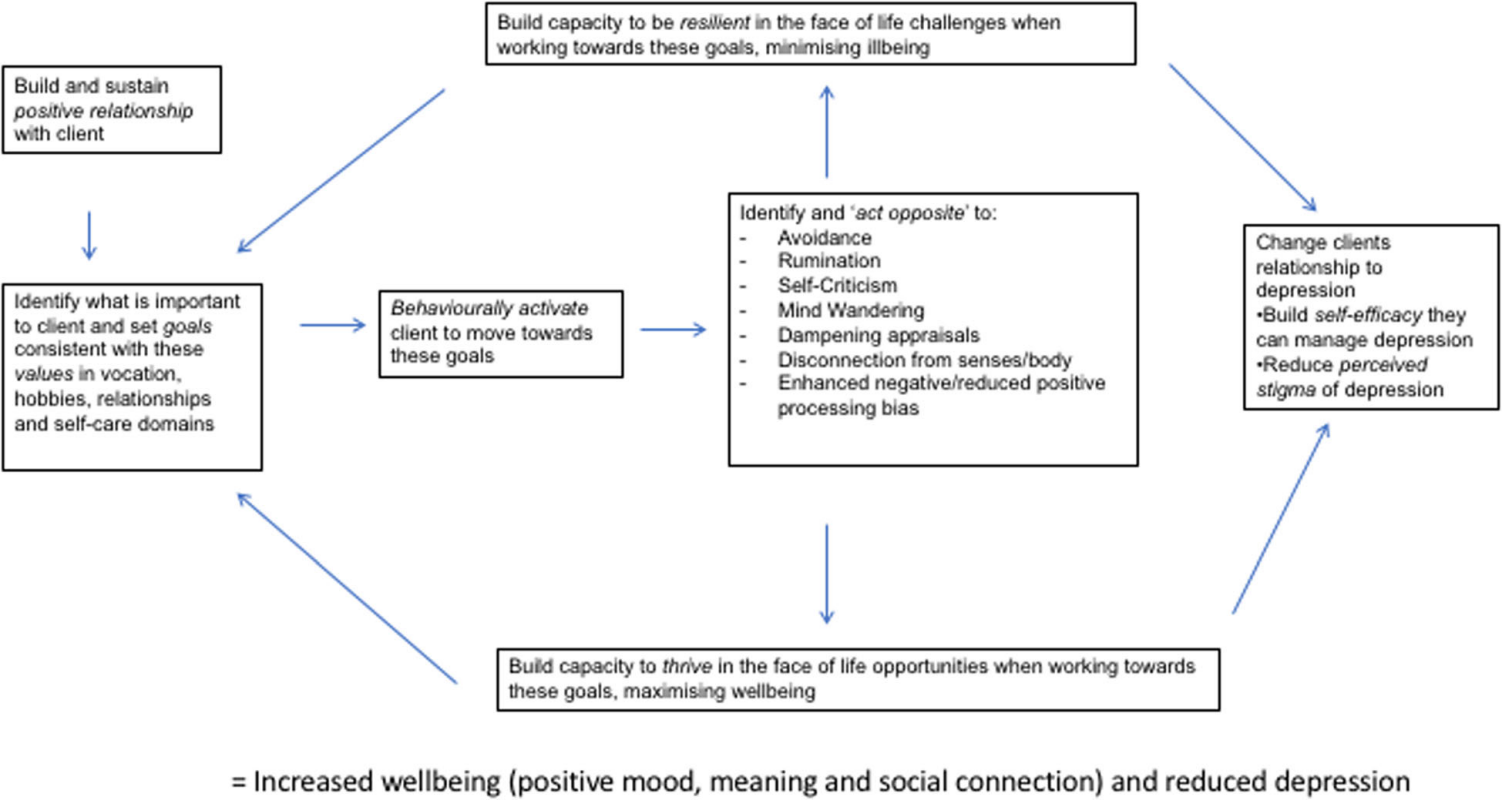
Logic model of ADepT

ADepT is a solution-focused, cognitively augmented, behavioural activation individual therapy approach, consisting of 15 acute treatment sessions and up to 5 booster sessions. Clients clarify what is important to them in vocation, relationship, hobbies, and self-care domains (their values); set behavioural goals consistent with these values; break these goals into steps; and systematically work towards completing these steps. Clients are encouraged to build the capability, opportunity, and motivation to carry out each action step [35]. Therapists support the client to understand how patterns of thinking and behaviour help and hinder them from fully taking opportunities to enhance positive mood (thriving) and overcoming challenges to minimise negative mood (being resilient)(see intervention description below for full details; see Fig. 1 for logic model of the approach). Given economic pressures on health care provision, ADepT was designed to be cost neutral in comparison to other established psychosocial treatments. If ADepT is cost neutral and leads to superior outcomes compared to existing treatments, then it would be the preferred approach. Taking into account the importance clients place on repairing anhedonia/wellbeing deficits to enhance recovery, improvements in wellbeing and reduction in depression symptoms were set as co-primary outcomes for ADepT.

We have conducted a case series evaluation of the ADepT treatment protocol on 13 currently depressed patients to preliminarily assess its feasibility, acceptability, and clinical efficacy [36]. All patients completed treatment and judged the therapy as acceptable. There were significant improvements in levels of wellbeing and reductions in anhedonia symptoms and depression symptoms, each of a large effect size according to Cohen’s rules of thumb [37]. A majority of participants showed reliable or reliable and clinically significant improvement, and no patients showed reliable deterioration on any of the outcome measures (cf., [38]). Follow-up assessments at 1 year indicate that these gains in ADepT were largely preserved. We benchmarked the case series outcomes against findings from the COBRA trial evaluating BA and CBT in the treatment of depression [39]. ADepT and both arms of the COBRA trial showed broadly comparable depression outcomes (all of a large effect size). However, anhedonia outcomes were superior for ADepT (a large effect size) compared to the (negligible) effect sizes found in both arms of the COBRA trial.

### Next steps in evaluating ADepT protocol

The ultimate ambition is to conduct a randomised controlled trial evaluating if ADepT is clinically effective and cost effective, relative to existing treatments such as CBT. However, uncertainties about the ADepT intervention remain and refinements may need to be made before proceeding to full trial. This pre-trial work includes further establishment of proof-of-concept, feasibility, and acceptability; refinement of the training and supervision pathway; development of a fidelity assessment tool; and estimation of likely cost and cost effectiveness. Uncertainties about trial design also need to be resolved, including the following: can participants be recruited to time and target; are clients willing to be randomised to CBT versus ADepT; is the measurement burden acceptable; are allocation, blinding, and health economics procedures fit for purpose; what primary outcome measure(s) should be used; and what counts as minimum clinically important difference on these measures (using this information to inform sample size calculation for a subsequent trial). The proposed pilot trial (aiming to recruit 80 depressed participants) will address these issues.

The following continuation criteria must have been met to proceed to definitive trial without modification:

1. Trial and ADepT treatment participation do not lead to serious negative consequences for participants (unexpected, clearly trial- or treatment-related serious adverse reaction).
2. All serious concerns about the acceptability, feasibility, and proof of concept of the ADepT treatment protocol and the trial design can be rectified.
3. At least 60% of the target sample size can be recruited.
4. At least 60% of participants in each treatment arm receive a minimum adequate dose of treatment (attend at least 50% of possible sessions).
5. Follow-up data on candidate primary outcome measures are available for at least 60% of the target sample size at 6, 12, and 18 months.

## Method

This protocol is reported according to the SPIRIT 2013 statement (see Additional file 1: SPIRIT figure; see Additional file 2 for a completed SPIRIT checklist).

### Aim

The aim of the study is to evaluate proof of concept, feasibility, and acceptability of ADepT and to evaluate the feasibility of a definitive randomised controlled design comparing ADepT to CBT in the treatment of depression.

### Design

This is a single-site pilot study, using a two-arm randomised parallel controlled trial design. Participants will be randomised using a 1:1 ratio to either ADepT or CBT. Outcome and health economics measures will be taken at baseline and at 6-, 12-, and 18-month post-randomisation (with 6 months as the primary end point). Quantitative self-report process evaluation measures will be taken at intake, 4 and 8 weeks into treatment, immediately post-treatment, and at 6 months. A qualitative evaluation will be undertaken at 6 and 18 months.

### Study setting

The trial will be conducted at the Accessing Evidence Based Psychological Therapies (AccEPT) clinic, University of Exeter, UK (a NHS commissioned out-patient service).

### Participants

Eligible participants will be aged over 18 years, meet criteria for a current major depressive episode based on a Structured Clinical Interview for Diagnosis (SCID-I) [40], and score in the clinical range of the Patient Health Questionnaire 9 (PHQ-9) [41] with marked anhedonic features (PHQ-9 total score of 10 or more; item one measuring anhedonia score of two or more). Participants will describe depression as their primary presenting problem and have sufficient knowledge of written and spoken English to be able to make use of the therapy and to complete research assessments without the need for a translator.

Exclusion criteria will include schizophrenia, bipolar disorder, learning disability, organic brain change, currently receiving any other psychosocial therapy, substance abuse that compromises ability to use therapy, current marked risk to self (self-harm or suicide) that cannot be safely managed in the clinic setting, and/or other significant conditions that mean the participant will not be able to participate in the trial (or will be put at risk by doing so).

Participants will have travel costs reimbursed and be given a £10 honorarium for each research assessment (intake and 6, 12, and 18 months) they complete (but not for attending each treatment session).

### Sample size

We aim to recruit 80 participants (40 per treatment) arm. Assuming a retention rate of 80% during the trial, this will lead to 32 treatment completers with complete data. This sample size will allow us to address the proof of concept and feasibility objectives of the pilot trial with sufficient precision and efficiency [42] (see Table 1).

**Table 1:** Precision/power of pilot trial size to address study aims

Feasibility aim	Description
Estimation of recruitment rate	Recruiting 80 individuals allows an estimation of recruitment rate from our primary recruitment source of 30% with a margin of error of ± 5.80%, of 20% with a margin of error of ± 5.07%, and of 40% with ± 6.20% (according to the 95% confidence interval). Assuming a 30% recruitment rate, this will require contacting approximately 270 clients. Assuming a 20% recruitment rate, this will require contacting 400 clients. Assuming a 40% recruitment rate, this will require approaching 200 clients.
Estimation of retention rate	Recruiting 80 participants will enable estimation of retention rate (as a percentage of patients randomised) of 80% with a margin of error of ± 8.77%, of 70% with a margin of error of ± 10.04%, and of 90% with a margin of error of ± 6.57 (according to the 95% confidence interval).
Estimation of rate outcomes	Sixty-four individuals being retained allows estimation of a sutained recovery rate of 60% with a margin of error of ± 12.00%, of 70% with a margin of error of ± 11.23%, and of 50% with a margin of error of ± 12.25% (according to the 95% confidence interval).
Effect size estimates in ADepT arm	According to Cohen’s rules of thumb, at 80% power in a paired sample *t* test, a sample size of at least 13 is required to detect a large effect size (*d* ≥ .8), a sample size of at least 32 is required to detect a medium effect size (*d* ≥ .5), and a sample size of at least 197 is required to detect a small effect size (*d* ≥ .2). Therefore, assuming expected levels of recruitment and attrition, these analyses are powered to detect a medium or large pre- to post-treatment change in the ADepT arm for candidate outcome and mechanism measures to inferentially test proof of concept.

### Randomisation, allocation concealment, and blinding

Participants will be randomly allocated to either the CBT or ADepT arm in a 1:1 ratio, stratified by symptom severity using published cutoffs on the PHQ-9 (< 19 versus ≥ 19) and antidepressant usage (currently taking antidepressants versus not) (cf. [39]). A computer-based system will allocate the first 24 participants on a truly random basis, with 12 participants being allocated to each arm using a pre-generated static list. For subsequent participants, the allocation will use a minimisation method in order to maximise the likelihood of balance in stratification variables across the two treatment arms. Randomisation will occur after baseline assessments.

Concealment will be ensured by the use of an externally administered password-protected trial website, set up and maintained by the UKCRC accredited Peninsula Clinical Trials Unit, independent of the trial. The trial manager (EW) and administrators of the AccEPT clinic (who are independent of the trial team) will be the only people aware of treatment allocation.

The intake assessment will be completed prior to randomisation, so blinding is not required at this stage. All researchers conducting 6-, 12-, and 18-month assessments will be blind to allocation.

### Recruitment

The primary method of recruitment will be from waiting lists held by high intensity Improving Access to Psychological Therapies (IAPT) services in Devon. If required, we may broaden our recruitment strategy by recruiting patients from general practices on the basis of record searches and seeking referrals from local mental health assessment teams and other local psychology therapy providers (including the AccEPT clinic hosting the research). Members of staff in each recruitment setting will identify and approach eligible patients and invite them to return a permission to contact form to the research team. If clients return the permission to contact form, a member of the research team will then invite them to attend an initial assessment at the AccEPT clinic, sending a detailed information sheet for clients to read in advance of this appointment. This initial assessment (conducted by EW) will describe the study in full, determine participant eligibility, and take written, informed consent. Ineligible participants or those who wish to not take part will have their care handed back to the service from which they were referred.

### Trial interventions

#### ADepT arm

We will provide a detailed description of the ADepT intervention protocol to enable replication, evidence synthesis, and wider implementation (cf. [29, 43]). ADepT is made up of novel intervention elements translated from basic science and also integrates intervention elements from a range of existing treatments, including CBT [24, 44, 45], Acceptance and Commitment Therapy [46], Positive CBT [47], Strengths-Based CBT [48], Wellbeing Therapy [49], Goal-Setting and Planning (GAP) approaches [50], Behavioural Activation [51], Mindfulness-Based Cognitive Therapy [52], Future Directed Therapy [53], the Cognitive Behavioural Analysis System of Psychotherapy [54], and Dialectical Behaviour Therapy (DBT) [55]. ADepT also integrates ideas from the positive psychology literature [56, 57].

The acute phase of ADepT consists of up to 15 individual therapy sessions (delivered approximately weekly and aiming to be completed within 6 months). As depression often has a chronic relapsing course, to help sustain long-term wellbeing, an additional five optional booster sessions (scheduled flexibly over the year following acute treatment) are offered to clients. This means treatment in total can be of up to 20 sessions across an 18-month period. Each session will last 60 min, with the exception of the initial assessment which can last up to 90 min. Sessions will typically be delivered face-to-face in a therapy room, although at times may be conducted over the telephone.

To overcome the pervasive, negatively biased information processing that characterises depression, ADepT therapists will be trained to adopt a positive, solution-focused style, ‘thickening the positive narrative’ wherever possible when clients exhibit pockets of resilience and thriving. The intention is to build an alternative positive information processing habit [58, 59], developing, generalising, and sustaining positive ways of thinking in clients [47, 48, 60]. The focus in ADepT is primarily on the ‘here and now’ and the future [53, 61]. Clients are supported to develop a positive, recovery-oriented relationship to their depression [62], seeing it as something that they can learn to manage well so that it does not get in the way of living the life they wish to lead. There is an emphasis on turning depressed clients into their own environmental change agents, building a positive, reinforcing communication style to enable clients to shape their work, hobbies, and relationships and to skilfully seek social support from others.

Given increasing evidence that therapy outcomes are improved if treatment is memorable, therapists will use techniques to enhance memorability of sessions (attention recruitment, categorisation, evaluation, application, repetition, practising remembering, use of cue-based reminders, and praising recall) [63, 64]. Clients will also be given handouts summarising the key learning points covered in each session and explaining how to use the ADepT tools. This is to help clients consolidate learning from each session (and also to ensure they have resources in the future if they wish to revisit what was covered in session).

Each session will utilise the following structure: conducting a mood review, setting an agenda, reviewing homework, working through each agenda item, and then asking the client to summarise and give feedback on the session. Home practice and exercises will be set between sessions. Therapists will actively seek and act on feedback from clients about how to make sessions most useful. Table 2 provides a breakdown of the approximate content covered in each session (although therapists will be encouraged to tailor when and whether different elements are introduced to match client needs).

**Table 2:** Session by session content in ADepT intervention

Session	Description
1	Assess the clients’ depression, review what is currently helping and not helping about how they are managing it, and introduce the ADepT rationale and structure. *Home activities:* Read treatment rationale handout, watch ‘BlackDog’ video about living with depression, and complete mood diary
2	Review mood diary and reaction to rationale and video; identify values in vocational, relationship, self-care, and leisure domains; and introduce to ‘dartboard’ exercise. This involves rating how close to the ‘bullseye’ behaviour is to key values in each life domain. *Home activities:* Read values handout, refine values, and complete mood diary
3	Review mood diary and values homework, set values consistent goals in each life domain using extended ‘dartboard’ exercise. *Home activities:* Read goals handout, refine goals, and complete mood diary
4	Review mood diary and goals handout, use a goal planning and monitoring tool to break goals down into SMART action steps, and build the capability, opportunity and motivation to carry out each action step. *Home activities:* Read goal planning and monitoring tool handout and use to address one goal, read handout about overcoming snags that block goal pursuit
5	Review use of goal planning and monitoring tool. Introduce to mapping tool, which formulates mechanisms that help/hinder resilience/thriving. This tool can be used to map out an ‘old me’ (depressive coping) and to develop a ‘new me’ (constructive coping) in a given situation. The ‘new me’ formulation will be utility based, focusing on what the goal is in a given situation and then what would be a way of thinking and behaving that would be most likely to bring this about. *Home activities:* Use mapping tool to analyse one opportunity and one challenge in the next week
6	Review use of mapping tool. Introduce to positive diary keeping to capture moments of resilience and thriving. This intends to build a positive, specific memory, and attentional style. *Home activities:* Read handout on positive diary, complete positive diary for next week
7	Introduce to mindful engagement with everyday wellbeing activities that enhance pleasure, meaning, and social connection. *Home activities:* Read handout on everyday wellbeing activities, practice mindful engagement with everyday activities, and continue to complete action steps
8 to 12	Use above tools to work through action steps identified above and develop new ways of coping when engaging with opportunities and challenges (‘acting opposite’ to depressive mechanisms). This will consist of psychoeducation around mechanisms, skills training around alternative ways of coping, and conducting behavioural experiments to test out and refine these new ways of coping. *Home activities:* Dependent on client goals and learning needs
13 to 15	Develop wellbeing plan to continue to build wellbeing in months after therapy. This can include reviewing goal progress and setting future goals on ‘dartboard’, reviewing key mechanisms helping/hindering resilience/thriving on formulation tool, reviewing key therapy techniques using a checklist, formulating a ‘relapse signature plan’ (early warning signs mood is dropping and steps that will be taken to minimise this), formulating a ‘wellbeing signature plan’ (early indicator signs mood is lifting and steps they will take to maximise this), and sustaining engagement with everyday wellbeing activities and ‘positive review’. If useful, a carer/partner can be invited into later ADepT sessions to share learning and support the client with ongoing change after acute therapy has completed. *Home activities:* Read and complete wellbeing planning tools
16 to 20	Five optional booster sessions will then be offered over the year after therapy. These will be used to review progress with goals, celebrate success, and troubleshoot any difficulties. *Home activities:* Dependent on client goals and learning needs

A bespoke ADepT training manual has been developed for the purposes of this trial. Therapists will attend an initial 1-day training workshop to familiarise themselves with the rationale of the approach, to learn key therapy principles, and to develop an ADepT therapeutic style. They will then be given detailed notes describing the aims and content of each session, with example audio clips from sessions with previous clients to illustrate ways of working (a detailed paper describing the ADepT protocol and presenting some case examples is to follow). Therapists will also be encouraged to practice ADepT techniques on themselves. Therapists will be recruited who have background in CBT (including clinical psychologists, nurses, and other high intensity trained therapists from Improving Access to Psychological Therapies Services). ADepT supervision will be provided in a small group format of 90 min weekly. Supervision and training will be provided by the project principal investigator (BD: an accredited CBT therapist and developer of the ADepT approach). Therapy competence and adherence to the ADepT protocol will be assessed with a bespoke fidelity rating tool.

#### CBT

CBT will consist of up to 20 (approximately weekly) individual therapy sessions over a 6-month period, following the CBT protocol developed in the COBRA trial [39, 65]. This protocol is predominantly based on the original Beckian CBT [24], updated to manage treatment-resistant depression [44] and to use a strengths-based formulation approach [45]. CBT focuses on engaging clients with pleasant activities and then identifying and modifying patterns of negative thinking that maintain the depressed mood. Sessions will again last up to 60 min each, supplemented by home practice between sessions. A 1-day top-up training will be provided to orient therapists to the trial and to familiarise themselves with the CBT protocol. CBT supervision (by accredited CBT therapists with experience in supervision of this protocol) will be provided in individual or small group format (up to 90 min per week as required). As in the ADepT arm, psychological therapists with a background in CBT will be recruited to deliver CBT in the trial and therapist competence and adherence will be measured using a fidelity rating tool.

### Clinical outcome assessments

Assessments will be conducted face-to-face or over the telephone at intake and 6, 12, and 18 months post-baseline. All participants will be invited to complete these assessments, irrespective of whether they deviate from the treatment protocols. The candidate co-primary outcome measures are the 9-item PHQ-9 [41] to measure depression symptoms and the 14-item Warwick-Edinburgh Mental Wellbeing Scale (WEMWBS) [66] to assess wellbeing. Changes in anhedonia and positive affect (key features of depression that ADepT specifically targets) will be measured using the 20-item Positive and Negative Affect Schedule (PANAS) [67] past-week version, the 14-item Snaith-Hamilton Pleasure Scale (SHAPS) [68], and the 10-item anhedonic depression subscale of the 30-item Mood and Anxiety Symptoms Questionnaire (MASQ-D30) [69]. Participants will also complete the 7-item Generalized Anxiety Disorder Scale (GAD-7) [70] to measure anxiety symptoms and the 10-item anxious arousal and 10-item general distress subscales of the MASQ-D30 [69]. The researcher will administer the Structured Interview Guide for the Hamilton Depression/Anxiety Rating Scales (SIGH-D and SIGH-A) [71, 72] to assess anxiety and depression severity and the Structured Clinical Interview for Depression (SCID) [40] to assess current depression diagnostic status. At 12- and 18-month assessments only, the Longitudinal Interval Follow-up Evaluation (LIFE) [73] will be used to assess depression diagnostic status over the previous 6-month period.

Complying with minimum data set requirements for Improving Access to Psychological Therapy (IAPT) services, immediately prior to each therapy session, participants in both arms will also complete the PHQ-9 [41], GAD-7 [70], IAPT phobia scale [74], Work and Social Adjustment Scale [75] to measure functional impairment, and IAPT employment questions to assess employment status. Given the anhedonia/wellbeing focus of ADepT, all participants will additionally complete the short form of the PANAS past-week version [76] as a measure of positive and negative affect, and the short (seven-item) version of the WEMWBS [77]. These weekly measures will make it possible to examine the trajectory of change on each outcome during acute treatment, informing when process evaluation mechanism of change measures should be optimally administered in a subsequent definitive trial. Successful acquisition of outcome data prior to every treatment session in routine IAPT service settings in the UK National Health Service indicates that it is feasible to collect weekly data in this way.

### Health economic outcome assessment

The health economics will take a NHS and personal social services perspective. Participants will complete the following health economic measures at baseline and at 6-, 12-, and 18-month follow-up: the Adult Service Use Schedule (AD-SUS)(see [78, 79]) to capture health/social care services used, the absenteeism and presenteeism items of the World Health Organisation Health and Performance Questionnaire (HPQ) [80] to index productivity losses, the EuroQol five dimensions questionnaire (EQ-5D-5L) [81] as a health-related quality of life index, and the ICEpop Capability measure for Adults (ICECAP-A) [82] as a wellbeing-related quality of life index. Socio-economic status will be measured by assigning participants an occupation code [83] and asking them about employment status, the size of the organisation they work in (if appropriate), and if they have supervisory experience (if appropriate). We will examine the utility of a resource log (a diary that participants can fill in each time they make use of services to serve as a memory aid) [84] to help capture service use in the definitive trial. The first 40 participants will be given a resource log at baseline, and the second 40 participants will not be given the resource log, allowing us to examine whether the resource log enhances the capture of health service utilisation data. Information on the resource use and costs of delivering the ADepT and CBT treatments (including sessions attended, cancelled, and DNAs; amount of time spent training and in supervision) will be collected.

### Treatment fidelity assessment

With participants’ consent, all sessions will be audio recorded. A random sample of up to 20% of all tapes, stratified by therapist, therapy session, and intervention, will be assessed by independent raters for competence rating using the Cognitive Therapy Scale-Revised [85] for the CBT arm and the bespoke ADepT supervision rating tool for the ADepT arm.

### Quantitative process evaluation

Consistent with MRC guidance about how to conduct process evaluations of complex interventions [86], a series of self-report questionnaire measures will be administered at baseline, after session 4, after session 8, after the final acute treatment session, and at 6-month follow-up to test the a priori specified ADepT logic model of change. To assess if clients have identified and are working towards value consistent goals, participants will complete a bespoke 20-item values rating scale developed for the purposes of this study (ADepT values rating scale; AVRS). The 12-item Savouring Beliefs Inventory (SBI) [87] will assess the capacity to thrive. The 10-item Connor-Davidson Resilience Scale (CD-RISC 10) [88] will assess resilience. The 10-item General Efficacy Scale (GSE) [89] will assess self-efficacy. The 10-item Internalised Stigma of Mental Illness Scale (ISMI-brief) [90] will assess clients’ relationship to their depression. To assess the quality of the therapeutic alliance, participants and therapists will complete the 10-item Working Alliance Inventory-Short Revised Form (WAI-SR and WAI-SRT) [91] after session 4, session 8, and final acute treatment session.

Change in key mechanisms impacting on resilience and thriving will be assessed using the following scales: avoidance via the 9-item Behavioural Avoidance in Depression Scale (BADS-SF) [92], rumination via the 10-item Ruminative Response Scale of the Ruminative Responses Questionnaire (RSQ) [93], self-criticism via the 12-item Self-Compassion Scale Short Form (SCS-SF) [94], mind wandering via the 15-item Five Facet Mindfulness Questionnaire Short Form items on acting with awareness (FFMQ-SF) [95], dampening appraisals via the dampening scale of the Response to Positive Affect Scale (RPA) [96], and disconnection from sensory and bodily awareness via a bespoke 6-item Brief Awareness of Everyday Sensory Experiences (BASE) scale developed for the purposes of this trial.

To assess momentary affective experience in day-to-day life, participants will undergo the experience sampling method (ESM) at intake and 6 months only (not at 12- or 18-month follow up to minimise participant burden). ESM is a momentary assessment technique in which participants are prompted to report on their current experiences at multiple moments during the day. ESM is therefore ideally suited to investigate changes in people’s emotional reactions to their daily environment in a reliable, ecologically valid fashion that minimises retrospective memory bias [97]. Participants will be lent a phone and then asked to use a bespoke application to answer a series of questions at eight daily intervals (one prompt delivered at a quasi-random time within each 2-hour period of the waking day) in the week before treatment starts and for a week following the 6-month outcome assessment (adapting methodology used by [98]). At each time point, positive and negative mood will be assessed by average rating of experience of a series of positive and negative emotion states in the present moment, on a sliding visual analogue scale ranging from 1(not at all) to 100 (extremely). Participants will also answer a series of questions that assess their levels of wellbeing, resilience, and thriving and use of psychological mechanisms targeted by ADepT treatment, using the same visual analogue scale. A series of multiple response items will classify the nature of the activity the participant is completing at that time. ESM data have a hierarchical structure. Multiple observations (level 1) are clustered within participants (level 2). Multilevel linear regression analyses take the variability associated with each level of nesting into account [99] and will be used to analyse the ESM data.

**Fig. 2:**
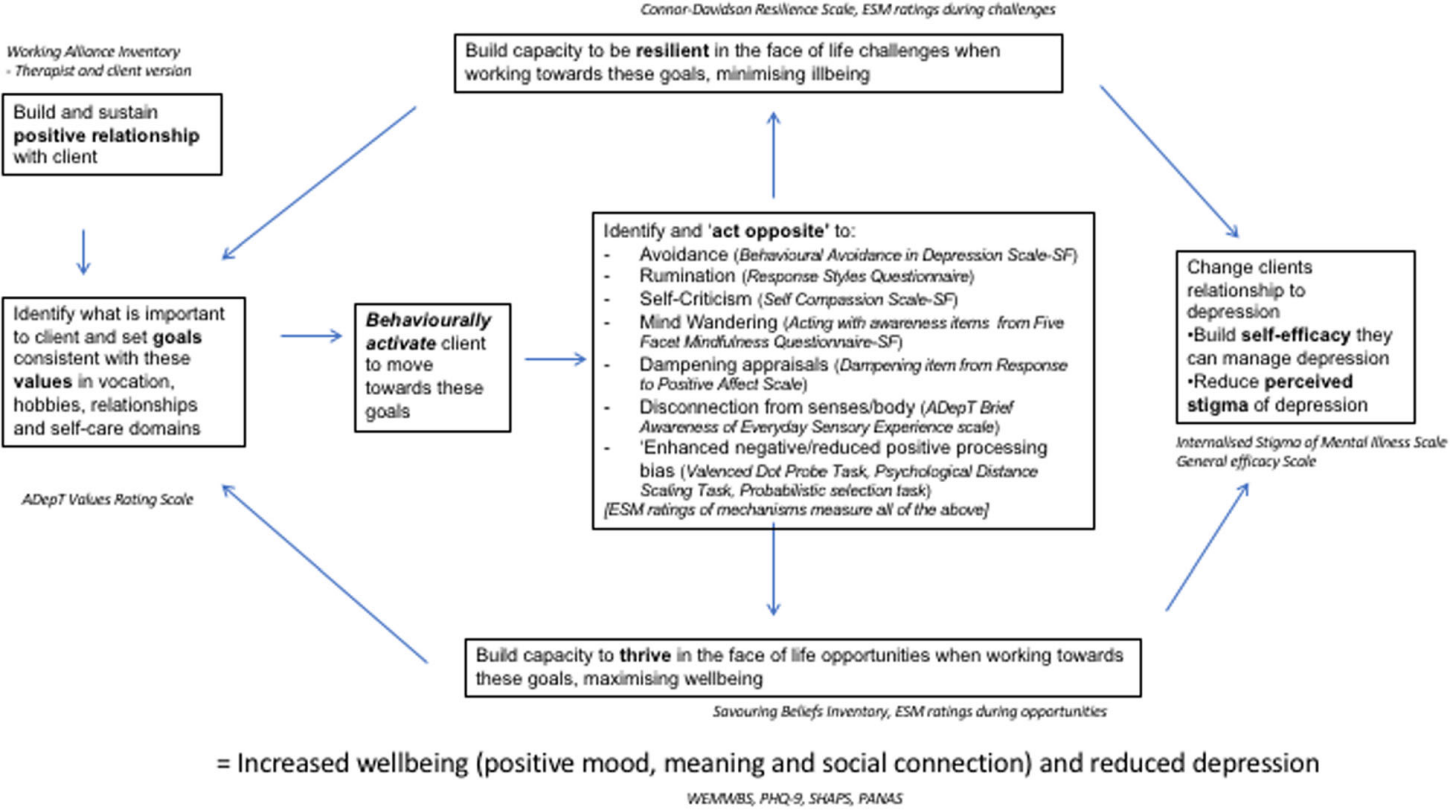
How process evaluation measures assess logic model of ADepT

The following laboratory experimental measures of mechanism of change will also be administered at baseline and 6 months only to assess positive versus negative information processing biases. A valenced dot probe task will be used to measure attentional bias to positive versus negative material (VDP) [100]. The Psychological Distance Scaling Task (PDST) [101, 102] will be used to measure positive versus negative schema organisation. The Probabilistic Selection Task (PST) [103] will be used to measure how quickly participants learn from positive versus negative reinforcement contingencies. Fig. 2 summarises how the various mechanism measures test the logic model of change.

To assess acceptability of the interventions, the following ratings will be taken. Following their first treatment session, participants in both arms will complete the six-item credibility/expectancy questionnaire (CEQ) [104] to gain their initial views on treatment. At 6 months, participants in both arms will be asked to rate the acceptability of the treatment they received (from 1 = not at all acceptable to 5 = very acceptable), how satisfied they were with the treatment they received (from 1 = not at all satisfied to 5 = very satisfied), and how likely they would be to recommend that treatment to friends or family if they needed similar care or treatment (from 1 = extremely likely to 5 = extremely unlikely). These questions will be repeated at 12 months, and participants in the ADepT arm will additionally be asked how useful they found the booster sessions on a scale (from 1 = not at all useful to 5 = very useful).

### Qualitative process evaluation

Patient and clinician experience and views on ADepT treatment and trial processes will be canvassed in a variety of ways, allowing us to further evaluate mechanisms of impact and also to explore implementation issues and any modifying influence of contextual factors (consistent with MRC guidance on process evaluation) [86]. This will help us understand the acceptability of the intervention and optimise its subsequent refinement, both in terms of running a future definitive trial but also in terms of whether ADepT could be implemented and sustained in routine NHS services in the future if it is shown to be clinically effective and cost effective.

A feedback booklet will be given to all participants at 6 months to allow them to provide brief written accounts of their experience of treatment and taking part in the research study. A subsample of participants will then be invited to a face-to-face qualitative interview, estimated to be 30 participants, or until data saturation has been reached. Recruitment will be purposive, seeking to achieve maximum variation in relation to (i) treatment arm (mixture of ADepT and CBT), (ii) completion/non-completion of treatment (based on minimum adequate dose of eight or more sessions), and (iii) response/non-response to treatment (based on a drop of PHQ-9 of 6 points or more during acute treatment). Subsampling will also be informed by participants’ written responses in the feedback booklet and will give us the opportunity to explore any unanticipated experiences and effects in more depth. Interviews will be conducted within 6 months of completing the feedback booklet (i.e. before the 12-month follow-up), will last approximately 45 min, and will explore participants’ experiences of the therapy and trial procedures at the end of acute treatment. The interviews will follow a topic guide to ensure that all areas are covered, but this will be used flexibly so as to allow other issues of importance to participants to be fully examined.

At 18-month follow-up, all participants in the ADepT arm will complete a further written feedback booklet, asking about their views on and experience of the booster sessions in ADepT.

All therapists and supervisors will also be invited to take part in a 45-min qualitative interview at the end of the trial to explore their views on treatments (including their views on feasibility, acceptability, efficacy, mechanism of change, and implementation) and on trial design. Due to resourcing constraints, both the quantitative and qualitative process evaluation will be conducted by the trial team rather than by independent researchers.

### Establishing minimum clinically important difference for outcome measures

To calculate anchor-based estimates of minimum clinically important difference (MCID) on the candidate outcome measures, at 6 and 18 months, participants will be asked to rate whether they feel that treatment led them to feeling very much better, much better, a little better, no change, a little worse, much worse, or very much worse in terms of their levels of depression symptoms, anxiety symptoms, wellbeing, anhedonia symptoms, and positive and negative mood (using a modified version of the Clinical Global Impressions Scale for each symptom class) [105]. These answers will be cross-matched to the degree of change that participant showed on each candidate measure to help establish mean and median amount of change that corresponds to each category. To calculate distribution-based estimates of MCID, we will calculate the standard deviation on each measure at intake (collapsing across treatment arms). Consistent with previous studies [106], we will use improvements of at least half a standard deviation to indicate clinically important change.

To capture participants’ views on outcome measures, at 18 months, participants will be given a copy of the measures to review and will then be asked to answer a series of questions about each of them in turn. First, participants will rate to what extent they agree with the statement that the measure captures what it is important to improve on during treatment for depression, on a scale from 1 (strongly agree) to 5 (strongly disagree). Second, participants will estimate clinically important change on that measure (defined as the smallest amount of change for them to feel the treatment had made a difference which was worth having treatment for). Third, participants will be given the opportunity to give narrative comments about what they think of that measure. Finally, participants will rank the measures from 1 (best) to 5 (worst) in terms of which is the best measure to use in the definitive trial to indicate whether treatment has been helpful (allowing them to use equal ranks if they wish).

## Analysis

### Quantitative analysis

Prior to quantitative analysis, data anonymization, checking and cleaning will be conducted and the database finalised by the trial manager (EW). The primary analyses will be conducted when the 6-month follow-up is complete (with further analyses at 12 months and 18 months). Feasibility analyses will use complete case data only. Inferential proof of concept analyses will be conducted on an intention to treat basis and will utilise multiple imputation to model missing data (only if assumptions regarding random missingness of data are met). Analyses will be conducted by the principal investigator (BD), who will remain blind to treatment condition until after the 6-month follow-up analyses have been completed.

#### Proof of concept analyses

All continuous (mean and SDs) and categorical (percentages) clinical outcomes and mechanism measures will be reported descriptively for each arm at each assessment point. We will report the proportion of participants in each arm showing reliable change and reliable and clinically significant change (cf. [38] at 6 months, 12 months, and 18 months, relative to baseline data, on each clinical outcome measure. We will use estimates of minimum clinically important difference derived from the trial to determine the cutoff for clinically significant change. Paired sample *t* tests will assess whether there is a significant improvement, relative to baseline, at each assessment point on each outcome measure and mechanism measure for participants in each arm. We will report the effect size for each of these analyses (Cohen’s *d*; Cohen, 1988; with 95% confidence intervals) and interpret them according to existing rules of thumb (< .2 = negligible, .2 to .5 = small, .5 to .8 = medium, and > .8 = large). To allow benchmarking against outcomes in routine NHS IAPT care, we will report the proportion of clients in each arm at 6-month assessment meeting IAPT criteria for reliable improvement (> 6 point reduction on the PHQ-9), recovery (PHQ-9 < 10 and GAD-7 < 8), and reliable recovery (meeting both reliable improvement and recovery criteria). To allow benchmarking with previous depression trials, we will also report the proportion of clients in each arm at 6-month assessment meeting criteria for response (> 50% drop in symptoms from intake) and remission (scoring beneath clinical cutoffs) on the PHQ-9 and HDRS. For the PHQ-9, the remission cutoff is scoring < 10, and for the HDRS, the remission cutoff is scoring < 8. For the subset of participants who no longer meet diagnostic criteria for a major depressive episode at 6 months, we will also report the proportion of individuals in each arm who show sustained recovery (i.e. do not meet diagnostic criteria at any time during the follow-up phase) at 12 months and 18 months. For those who relapse, we will report the mean time to relapse in each arm. We will also report the number of people showing a treatment-related serious adverse reaction in each arm.

#### Feasibility and acceptability of ADepT intervention

For each arm, we will report the number of participants who (i) withdrew from the trial prior to randomisation and indicated that this was because they could not receive their preferred treatment and (ii) who withdrew from the trial during active treatment and stated this was due to dissatisfaction with the treatment. Patient adherence to treatment will be indexed by the mean (SD) number of therapy sessions offered and attended and by calculating the proportion of participants who receive a minimum adequate dose (eight or more sessions) in each arm. To determine initial views on acceptability of each treatment, mean (SD) of session one CEQ [104] ratings will be reported. To determine post-treatment views on acceptability, participants mean (SD) ratings of treatment acceptability and satisfaction and whether they would recommend treatment to friends and family at 6 months and 18 months will be reported.

#### Refinement of fidelity ratings

To evaluate and refine how well procedures and tools rating therapy quality and adherence to protocol are functioning, we will assess the proportion of clients who give consent for sessions to be recorded and the proportion of sessions where the recording is captured in a way that can then be coded. We will assess the inter-rater reliability of this ADepT fidelity tool, selecting a random subset of session tapes in the ADepT arm to be rated by two experienced clinician raters. We will also ask experienced clinicians and supervisors to review the fidelity tool and listen to example tapes to set a cutoff for adherence i.e. the therapy is competently delivered, in a way that is adherent to the protocol and sufficiently differentiates it from CBT. We will revise the tool as appropriate.

#### Selecting outcome measures and informing sample size calculations

Mean (SD) patient rankings of each candidate outcome measure will be reported. Patient, clinician, and supervisor narrative views on each outcome measure will be thematically summarised. Minimum clinically important difference will be estimated for each candidate outcome by averaging findings from distribution, anchor, and patient report methods. The estimate of MCID and standard deviation at baseline of each measure will be combined to inform sample size calculations for a subsequent definitive trial (i.e. to calculate how many participants would be required to test MCID superiority of ADepT over CBT).

#### Informing recruitment for definitive trial

For each recruitment source, we will report the number of individuals identified, approached, consented, and randomised into treatment. We will also report the total number of participants randomised to treatment each month. For each arm, we will report the number of individuals completing acute treatment. For the ADepT arm only, we will report the number of individuals completing booster sessions.

#### Refining trial procedures for definitive trial

We will report the percentage of time researchers accurately guessed the blind (examining if this is significantly greater than chance) in each arm. Further, for each arm, we will report the number of times the blind was clearly broken, where the online randomisation system failed, where there were problems in subsequent allocation to treatment after randomisation, and where capture of outcome data failed (e.g. computer failure during experimental tasks), and the proportion of participants with complete data at each assessment point.

#### Health economic data

To estimate the costs of the two interventions for health economic purposes, resources involved in delivering CBT and ADepT will be collated directly from therapists (including training, supervision, and travel time). Intervention costs will be calculated via standard micro-costing (bottom-up) approaches, incorporating therapist salaries plus on-costs (pension/national insurance contributions) and appropriate capital, administrative, and managerial overheads. Descriptive data for each arm will be reported with 95% confidence intervals. To resolve uncertainties about health economic data completeness, we will test data collection procedures and record resource use categories prone to missing data/being misunderstood in self-report measures. To evaluate resource log utility, we will randomly sample a subset of individuals either given or not given the log and examine the degree to which resource use reported in the patient questionnaires matches medical records (via SUS or CRIS record search if available). These analyses will inform whether there is a need to develop strategies to collect missing data and/or to include proxy assessments in the main trial. We will use this health economic data, along with acute depression symptom and wellbeing improvements and 18 m relapse rates observed in this trial and other datasets, to develop the framework to model the likely cost-effectiveness of ADepT compared to CBT using decision tree modelling techniques [107].

### Qualitative analysis

Feedback booklets will be anonymised and interviews audio recorded, transcribed verbatim, and anonymised prior to analysis. Thematic analysis of both qualitative data sets will be conducted using a Framework approach, involving the coding and sorting of textual units according to both deductive and inductively derived categories, and the use of matrices to review the coded data, investigate commonalities and differences, and search for patterns [108, 109]. Initial coding and data management will be facilitated by NVivo software.

Qualitative analysis has the potential to clarify therapist and clients views about whether ADepT is an effective treatment and how it may work, to gain therapist and client views about the acceptability and feasibility of ADepT, to gain therapist views about the training pathway for ADepT and how this could be enhanced, to explore client views about which outcome measures best capture what is important to them for recovery, to explore therapist and client views about barriers and facilitators to recruitment into the trial, to explore client views about how taking part in the trial was experienced and whether the measurement burden was acceptable, and to explore client views about the utility of the resource log and any barriers or facilitators to accurate reporting of resource use.

### Exploratory condition analyses

While the study is not formally powered to detect condition differences, additional exploratory analyses will examine if there are any differences between the two treatment arms (all with condition as the independent variable, adjusting for stratification variables of depression severity and antidepressant medication status). Continuous outcome and process evaluation measures across treatment arms will be compared at 6-month, 12-month and 18-month assessments using ANCOVA analyses, adjusting for baseline outcome levels. We will report partial eta-squared as a measure of effect size (with 90% confidence intervals) for these ANCOVA analyses (see Steiger [110]). Time to relapse/recurrence between arms at 12-month and 18-month follow-up (in the subset of clients who recovered at six-months) will be compared using Cox regression proportional hazard survival analysis. Hierarchical linear modelling (growth curve analysis) on weekly outcomes during acute treatment will examine if the trajectory of change differs between arms. We will additionally explore whether the stratification variables (severity and medication status) are associated with condition effects, by entering interaction terms between condition and the stratification variables into analyses. Exploratory analyses will also be conducted on the laboratory mechanism measures (following analytic methods in existing studies using these tasks) and experience sampling measures (using as appropriate a hierarchical linear modelling approach to take into account the nested structure of the data). Finally, we will conduct exploratory Bayesian analyses to examine the expected additional benefit to new patients of being treated with ADepT rather than CBT on the two primary outcomes of PHQ-9 depression and WEMWBS wellbeing. We will report the 95% credibility interval of the difference between outcomes if new patients had been treated with ADepT rather than CBT at 6-month, 12-month, and 18-month assessment, based on a simulation from the primary outcome model (see Lynch et al. [111]).

## Trial management and governance

### Data management and storage

Clinical notes, measures, and therapy recordings will be stored according to the standard practice of the host AccEPT clinic. Hard copies of information/measures gathered as part of the research study will be anonymised and then stored in a locked filing cabinet in a locked office in the Mood Disorders Centre, University of Exeter. Consent forms will be stored separately from data. Data will be entered into an SPSS spreadsheet maintained by the trial manager (EW), stored securely on the University of Exeter server. The candidate primary outcome data [PHQ-9 and WEMWBS] will be double entered. Published material will not contain patient identifiable information. The datasets generated and/or analysed will be stored in a repository within the University of Exeter (and will not be made publicly available). Anonymised data may be accessed and analysed by members of the project team and with researchers collaborating with team members on the analysis of these data. With the exception of anonymised quotes from research interviews, consent from participants was not sought for sharing raw data publically. Therefore, external researchers who wish to access the data for future projects or analyses must do so via request to the principal investigator (or his delegate) and ensure necessary ethical and regulatory processes in the UK relating to the release of anonymised data have been followed.

Original research records will be retained for 7 years after the study end, after which time they will be kept in electronic form and the original records destroyed (including records of participants’ names and contact details). Audio files of participant interviews will be destroyed at this time (although transcriptions of the interviews will be kept in electronic form). The electronic records will be kept for at least 20 years after the study end.

### Study approvals

The study has received approval from the UK National Research Ethics Service (REC reference: 17/SW/0009) and the Health Research Authority (IRAS ID: 216871), and from all relevant local approval bodies.

### Anticipated risks and benefits

All participants recruited into the trial will receive up to 20 sessions of psychological therapy that is likely to be beneficial to their mood. During the active treatment phase of the trial, participants will be asked not to engage with any other psychological therapy (reflecting standard practice that only one therapy should be completed at a time), but no other aspects of standard care will be withheld as a result of trial participation. All participants in the trial will receive an enhanced level of monitoring by the research team. Individuals with depression are at increased risk of self-harm and suicide compared to the general population. The trial will follow established clinical and research protocols for monitoring and responding to self-harm, suicide risk, or other adverse events during therapy and research contacts. This may involve unblinding if required to ensure patient safety (asking the trial manager for treatment allocation).

### Trial governance

The University of Exeter will act as sponsor for this study. While not an essential requirement for a feasibility trial, we have convened a combined Trial Steering Committee and Data Monitoring and Ethics Committee (TSC/DMEC). This is independent from the sponsor; is made up of the principal investigator (BD) and trial manager (EW) and independent (academic, clinical, and lived experience) members; and will meet approximately four times over the life of the project. The terms of reference for the TSC/DMEC include monitoring trial progress, providing advice on scientific issues, and evaluating if there are any patient safety concerns that require the trial to be modified, paused, or discontinued. For all substantive changes to the protocol, approval will be sought from the sponsor and the relevant national regulatory bodies. As appropriate, trial registration will be updated.

### Patient and public involvement

The form and content of the ADepT intervention and the trial protocol was co-designed using input from the public and patient involvement lead on the project team (NR), other members of the Lived Experience Group (LEG) at the Mood Disorders Centre, University of Exeter (see http://www.exeter.ac.uk/mooddisorders/groups/leg//), and qualitative interviews with other service users. PPI members will also be involved in ongoing governance of the project, including NR meeting regularly with the PI and attending ADepT project meetings and another LEG member sitting on TSC/DMEC. We will follow national good practice guidelines for involving members of the public in research (see www.invo.org.uk.).

### Adverse events

In line with other complex intervention studies, we will monitor the occurrence of adverse events (any untoward or unintended medical occurrence or response whether it is causally related to the trial treatments or not). Adverse events will be classed as serious if they are fatal and life-threatening, require unplanned hospitalisation or prolong existing hospitalisation, result in significant disability or incapacity, or lead to any other condition judged significant by a clinician. Any serious adverse events that may be treatment-related will be reported to the TSC/DMEC. If the TSC/DMEC judge the event to be serious and treatment-related, the sponsor and NHS ethics committee will be informed immediately and a report sent to them within 14 days. If appropriate, the trial will be temporarily halted pending investigation and analysis of the extent to which future risk can be mitigated. If it is judged that this is not possible, the trial will be discontinued. The same process will be followed should information come to light that indicates that the therapy intervention or trial procedures are unsafe.

### Role of the funder and sponsor

The University of Exeter as a trial sponsor has ultimate responsibility over the study (contact: Gail Seymour; G.M.Seymour@exeter.ac.uk). The sponsor and the funder (the NIHR) have not been involved in the design of the study and will not have any significant role during the execution, analysis, interpretation, or publication of the study. The funder will be required to approve the final report prior to publication.

### Dissemination

Findings will be disseminated to participants, services, and other stakeholders at the local level through a written summary of results and public engagement events. Findings will be disseminated at a national and international level via conference presentations and where possible via media and social media. The dissemination strategy will be co-developed with public and patient involvement (PPI) input, including study co-applicant NR and LEG.

### Trial status

The trial was prospectively registered on ISTRCN on 27 March 2017 (10.1186/ISRCTN85278228). The trial opened to recruitment on 29 March 2017, and the first patient was randomised on 19 April 2017. Recruitment was completed by the end of July 2018. The final follow-up aims to be completed by the end of December 2019. Data analysis and reporting are expected to take a further 12 months.

## Discussion

This pilot trial is designed to assess the proof of concept, feasibility, and acceptability of Augmented Depression Therapy (ADepT) for individuals with depression.

The findings will inform future investigation of this approach, potentially via a definitive randomised controlled trial testing its clinical effectiveness and cost-effectiveness compared to CBT.

## Additional files

Additional file 1: ADepT SPIRIT figure (DOCX 25 kb)

Additional file 2: SPIRIT checklist (DOC 121 kb)

## Abbreviations

AccEPT: Accessing Evidence Based Psychological Therapy
ADepT: Augmented Depression Therapy
AVRS: ADepT values rating scale
BA: Behavioural activation
BADS-SF: Behavioural Avoidance in Depression Scale-Short Form
BASE: Brief Awareness of Sensory Experiences Questionnaire
CBT: Cognitive Behavioural Therapy
CD-RISC: Connor-Davidson Resilience Scale
CEQ: Credibility/expectancy questionnaire
DMEC: Data Monitoring and Ethics Committee
EQ-5D-5 L: EuroQol five dimensions questionnaire
FFMQ-SF: Five Facet Mindfulness Questionnaire Short Form
GAD-7: Generalised Anxiety Disorder 7 questionnaire
GSE: General Self-Efficacy Scale
HPQ: Health and Performance Questionnaire
IAPT: Improving Access to Psychological Therapies
ICECAP: ICEpop CAPability measure for Adults
ISMI: Internalized Stigma of Mental Illness Scale
LEG: Lived Experience Group
LIFE: Longitudinal Interval Follow-Up Evaluation
MRC: Medical Research Council
NIHR: National Institute of Health Research
NVS: Negative valence system
PANAS: Positive and Negative Affect Schedule
PHQ-9: Patient Health Questionnaire 9 questionnaire
PI: Principal investigator
PPI: Patient and public involvement
PVS: Positive valence system
RPA: Response to Positive Affect Scale
RSQ: Ruminative Responses to Depression Questionnaire
SBI: Savouring Beliefs Inventory
SCID: Structured Clinical Interview for Depression
SCS-SF: Self-Compassion Scale Short Form
SHAPS: Snaith-Hamilton Pleasure Scale
SIGH-A: Structured Interview Guide for the Hamilton Anxiety Rating
SIGH-D: Structured Interview Guide for the Hamilton Depression Rating
TAU: Treatment as usual
TSC: Trial Steering Committee
VDP: Valenced dot probe
WAI-SR: Working Alliance Inventory-Short Revised Form
WEMWBS: Warwick-Edinburgh Mental Wellbeing Scale
