# Supplementary material for: Evaluating Augmented Depression Therapy (ADepT): study protocol for a pilot randomised controlled trial
Source: Pilot Feasibility Stud. 2019 Apr 27;5:63. doi: 10.1186/s40814-019-0438-1 (PMC6486988; doi:10.1186/s40814-019-0438-1)
Supplement: Supplementary file 1 — ADepT SPIRIT figure (DOCX 25 kb) [file 40814_2019_438_MOESM1_ESM.docx]

ADepT SPIRIT Figure

|  |  | **STUDY PERIOD** | | | | | | | |
| --- | --- | --- | --- | --- | --- | --- | --- | --- | --- |
|  | **Enro-lment** | **Alloc-ation** |  | **Post-allocation** | | | | | **Close-out** |
| ***TIMEPOINT*** | ***-t_1_*** | **0** | ***t_1_*** | ***t_2_*** | ***t_3_*** | ***t_4_*** | ***t_5_*** | ***t_6_*** | ***t_7_*** |
| ***ENROLMENT:*** |  |  |  |  |  |  |  |  |  |
| ***Eligibility screen, consent and informed consent*** | X |  |  |  |  |  |  |  |  |
| ***Allocation*** |  | X |  |  |  |  |  |  |  |
| ***INTERVENTIONS:*** |  |  |  |  |  |  |  |  |  |
| ***ADepT Intervention*** |  |  |  |  |  |  |  |  |  |
| ***CBT Intervention*** |  |  |  |  |  |  |  |  |  |
| **ASSESSMENTS:** |  |  |  |  |  |  |  |  |  |
| **Clinical Outcome Assessments:** |  |  |  |  |  |  |  |  |  |
| - PHQ-9; GAD-7; PANAS; WEMWBS; SHAPS; MASQ-D30; SIGH-D; SIGH-A | X |  |  |  |  |  | X | X | X |
| - LIFE interview |  |  |  |  |  |  |  | X | X |
| **Health Economic Evaluation:**   - AD-SUS, HPQ, EQ-5D-5L, ICECAP-A | X |  |  |  |  |  | X | X | X |
| **Quantitative Process Evaluation:**   - CEQ |  |  | X |  |  |  |  |  |  |
| - WAI-SR, WAI-ST |  |  |  | X | X | X |  |  |  |
| - AVRS, SBI, CDI-RISC 10, GSE, ISMI-brief, BADS-SF, RSQ, SCS-SF, FFMQ-SF, RPA, BASE | X |  |  | X | X | X | X |  |  |
| **Quantitative Process Evaluation:**   - ESM, VDP, PDST, PST | X |  |  |  |  |  | X |  |  |
| - acute treatment acceptability ratings |  |  |  |  |  |  | X |  | X |
| - booster acceptability ratings (ADepT arm only) |  |  |  |  |  |  |  |  | X |
| **Qualitative Process Evaluation:**   - booklets and interviews |  |  |  |  |  |  | X |  | X |
| **MCID assessment:**   - questionnaire ratings and modified CGIs |  |  |  |  |  |  | X |  | X |

**Notes:** -t1=enrolment; t0=allocation; t1=first session of acute therapy; t2=4wks into acute therapy; t3= 8wks into acute therapy; t4=end of acute therapy; t5= 6-month follow-up; t6=12-month follow-up assessment; t7=18-month follow-up assessment and end of ADepT booster treatment. The intention is for acute treatment to have complete by the 6-month follow-up assessment, but it is possible this may not always be the case (i.e. t5 occurs before t4 for a subset of participants). ADepT and CBT acute treatment phase = solid arrow; ADepT booster phase = dashed arrow. During acute and booster treatment, the PHQ-9, GAD-7, IAPT phobia-scale, WSAS, PANAS-SF, and WEMWBS-SF are administered prior to each (approximately weekly) treatment session.
